# Nanomaterials in osteoarthritis therapy: advances in drug delivery, tissue regeneration, and implant engineering

**DOI:** 10.3389/fmed.2026.1775067

**Published:** 2026-03-26

**Authors:** Deliang Cheng, Qian Wang, Jiafeng Long, Xuehai Ou, Shaoyan Shi

**Affiliations:** Department of Hand Surgery, Honghui Hospital, Xi’an Jiaotong University, Xi’an, China

**Keywords:** biocompatibility, clinical translation, nanomaterials, orthopedic nanotechnology, osteoarthritis

## Abstract

Osteoarthritis (OA) is a progressive degenerative joint disorder characterised by cartilage destruction, subchondral bone alterations, and chronic inflammation. Existing therapeutic interventions primarily provide short-term relief but fail to repair damaged tissues. Nanotechnology has emerged as a potential solution to these shortcomings by facilitating targeted drug delivery, enhancing tissue regeneration, and improving implant performance. Nanomaterials such as metallic, ceramic, polymeric, carbon-based, and composite nanoparticles possess certain unique properties, including high surface area, tunable properties, and strong biological interactions, which aid cartilage repair, bone regeneration, and controlled release of therapeutics. These nanomaterials can cross thick cartilage matrices, protect drugs from degradation, and regulate inflammatory and oxidative pathways that drive the progression of OA. Additionally, nanocoatings improve implant osseointegration and inhibit infection. Nanocarriers encapsulate DNA, siRNA, mRNA, and other nucleic acids, protecting them from degradation and enabling precise delivery to diseased cells within the joint (e.g., chondrocytes or synovial cells) via surface modifications (e.g., ligands that target the cartilage matrix or specific cell receptors). Although the results are promising, challenges in safety, biodistribution, large-scale production, and regulatory approval remain obstacles to clinical translation. This mini-review primarily focuses on nanomaterial-based drug delivery, regenerative repair, and implant engineering strategies, while briefly discussing emerging nanomaterial–cell interactions where relevant. Nanomaterials have the potential to revolutionize the therapeutic management of osteoarthritis, but continued innovation and interdisciplinary collaboration are necessary to realise this potential.

## 1 Introduction

Osteoarthritis (OA) is the most prevalent degenerative joint disorder that afflicts millions of people around the globe and is a major cause of disability in the ageing population (1). It is characterised by disruption of the integrity of the articular cartilage and subchondral bone, synovial inflammation, and joint dysfunction. Although it is very prevalent and burdensome in terms of socioeconomic aspects, the current clinical therapies, such as nonsteroidal anti-inflammatory drugs (NSAIDs), corticosteroid injections, hyaluronic acid visco supplementation, and surgeries, do not focus on stopping or reversing the development of the disease, but rather alleviate pain and enhance mobility (2). The problem with these conventional therapies is that they tend to have short residence times in the joint, shallow penetration into dense cartilage, and no specific effect on pathological tissue (3). This underscores the need for new therapeutic approaches that can combat the biological processes underlying OA and facilitate true tissue restoration (4). Nanotechnology has emerged as an effective and promising approach to overcoming these constraints. Nanomaterials are materials with a size of 1–100 nm, which have specific features of physicochemical and biological characteristics that enable them to be close to cells, proteins, and other elements of the extracellular matrix (5). They have high surface area, tunable morphology, and are capable of delivering and releasing drugs in a controlled manner, making them highly suitable for orthopedic applications (6). In OA therapy, in particular, nanomaterials have the potential to deliver therapeutic molecules more efficiently to chondrocytes, synoviocytes, or sites of inflammation than conventional drug preparations, protect therapeutic molecules against rapid degradation, and penetrate cartilage tissue (7). Moreover, most nanomaterials, such as hydroxyapatite, bioglass, graphene oxide, and some metallic nanoparticles, have intrinsic bioactive, osteogenic, or anti-inflammatory capabilities that promote tissue regeneration (8). Moreover, nanotechnology has driven significant advances in bone regeneration, cartilage repair, and implant surface engineering. Nanostructured scaffolds combine the structure of natural bone and cartilage and stimulate cell adhesion, growth, and differentiation (9). Nanocoatings on orthopedic implants enhance processes in the event of osseointegration, wear, and infection prevention. Together the above developments underscore the potentials of nanomaterials to transform the future of treating osteoarthritis and orthopedics (10). This mini review elaborates the uses and benefits of nanomaterials in orthopedics, nature of nanomaterials used, their key therapeutic uses in osteoarthritis, and finally, an analysis of challenges and future research directions is made.

## 2 Role and advantages of nanomaterials in orthopedics

Modern orthopedics has a great role to play in the presence of nanomaterials due to their distinct nanoscale characteristics that are highly similar to the natural structure of the bone and cartilage (11). Being extremely small, having high surface area, and tunable physicochemical properties, they possess strong interactions with biological tissues and are therefore ideal for providing treatment to complex orthopedic diseases like osteoarthritis (12). Among the most important benefits of nanomaterials is their ability to enhance drug bioavailability and targeting (75). The conventional osteoarthritis medicines cannot enter cartilage easily, given its thick extracellular matrix, but nanoparticles can easily penetrate cartilage and reach chondrocytes, synovial macrophages and inflamed tissues with therapeutic molecules, given that it can easily diffuse through the network (13, 14). Nanocarriers minimize systemic toxicity and increase therapeutic efficacy by eliminating the fast degradation of drugs and prolonging and controlling the release of drugs (15). Besides enhanced delivery, nanomaterials offer mechanical and structural support because most of them resemble the natural bone mineral or collagen fibres. Nanofibers, nanotubes, and nanocomposite scaffolds provide a biomimetic environment that facilitates cell adhesion, proliferation, and differentiation and enhances bone and cartilage regeneration (16).

The other essential benefit is the inherent biological activity of some nanoparticles (17). Cerium oxide nanoparticles are among the materials used as antioxidants that counteract reactive oxygen species, which contribute significantly to the degeneration of cartilage (18, 19). Nano-particles of gold also have an anti-inflammatory effect, whereas silver and zinc oxide nanoparticles have strong antimicrobial effects, which are applicable in postoperative orthopedic surgery for the prevention of infections (20). Such biological activities assist in minimizing inflammation, slowing down the progression of osteoarthritis, and improving tissue protection (21). Many nanomaterials, particularly polymeric and ceramic nanoparticles, exhibit high biocompatibility and controlled biodegradability and at regulated intervals. This eliminates concerns about long-term accumulation and is appropriate for chronic conditions that require long-term care (22, 74). They also have a high surface area, which enables efficient loading with drugs, growth factors, genes, or targeting ligands, and multifunctional nanoplatforms can be developed that are capable of simultaneous therapy, targeting, and imaging (23). Besides, nanomaterials have synergistic physical and biological benefits. Examples include directing magnetic nanoparticles by external magnetic fields to different places in the joints and using carbon-based nanomaterials like graphene and carbon nanotubes to greatly improve scaffolds mechanical strength in bone repair or cartilage repair (24). Nanostructured surfaces such as titanium dioxide are used to enhance the bioassembly of implants, enhance the bone-implant interface, and inhibit inflammation (25). Microbial adhesion and biofilm formation can be prevented by the incorporation of antimicrobial nanomaterials like silver nanoparticles on the surfaces of the implants to combat one of the most significant complications of the orthopedic surgeries (26). In general, the structural, mechanical, biological, and drug-delivery benefits of nanomaterials are all exhibited in a unique combination, which makes them a more transformative tool in orthopedic treatment that has proven to deliver more effective, targeted, and long-lasting benefits as compared to traditional materials (27, 28).

## 3 Types of nanomaterials used in orthopedic therapy

**Figure 1:**
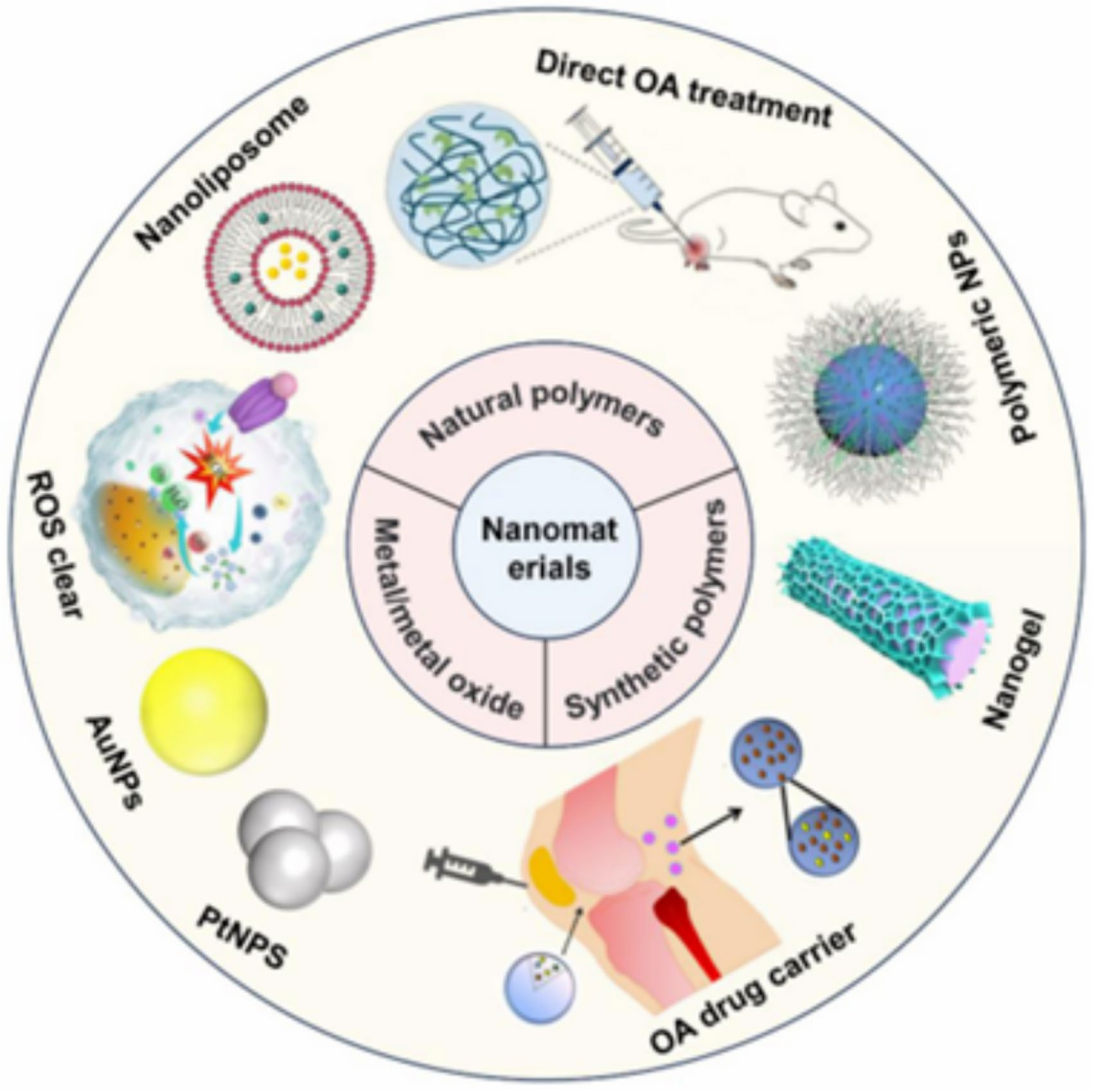
Schematic illustration of nanomaterial-based strategies for osteoarthritis treatment. Nanoliposomes, polymeric nanoparticles, nanogels, gold nanoparticles, and reactive oxygen species-scavenging nanomaterials are used to enhance targeted drug delivery, modulate inflammatory and immune responses, and promote cartilage and subchondral bone regeneration. These nanoplatforms regulate the joint microenvironment and support regenerative processes by interacting with resident cells, including chondrocytes and synovial cells. Reprinted with permission from (72) © 2025, © Deng W, Wang T, Li L, Xiao X, Xu Y, Li Q, et al. 2025. Published by Oxford University Press.

Orthopedic therapy is associated with a vast range of nanomaterials, and each of them possesses distinct structural, mechanical, and biological properties that predetermine their application in the treatment of osteoarthritis and other musculoskeletal problems (29). This number describes the main categories of nanomaterials used in the treatment of osteoarthritis, including natural polymers, synthetic polymers, and metallic or metal-oxide nanoparticles (30–34). Figure 1 illustrates the use of various nanosystems (nanoliposomes, polymeric nanoparticles, nanogels, AuNPs, and ROS-scavenging particles) as drug-delivery systems or as potential therapeutic agents. When combined, these nanomaterials enhance targeted delivery, reduce inflammation, and promote tissue repair in the treatment of OA.

### 3.1 Metallic nanomaterials

One of the most widely researched types of nanomaterials is metal nanoparticles, which include gold (Au), silver (Ag), magnesium-based nanoparticles, and titanium dioxide (35). These materials are prized for their high stability, surface-modification capacity, and inherent biological activities (36). Gold nanoparticles have been used as anti-inflammatory carriers and gene-delivery vehicles (37), whereas silver nanoparticles are commonly used as implant coatings due to their high antimicrobial activity and anti-inflammatory properties in the joint post-surgically (37). Nanomaterials composed of magnesium and titanium are also associated with enhanced osteogenesis and implant incorporation, as they are highly biocompatible and can stimulate bone cell activity (38).

### 3.2 Ceramic nanomaterials

Another significant type is ceramic nanomaterials, which are bioactive, osteoconductive, and resemble natural bone mineral. They are hydroxyapatite (HA), bioglass nanoparticles, zirconia, and alumina (39). The nanoparticles of Hydroxyapatite are very similar to the mineral part of the bone, and hence they are very important in bone restoration, subchondral bone repair, and scaffold formation (40). Bioglass nanoparticles are very bioactive and release favourable ions such as calcium and silicon that induce osteoblast differentiation and angiogenesis- important in the repair of osteoarthritic bone tissue (41). Ceramic nanomaterials can also serve as carriers for growth factors and drugs, thereby enhancing therapeutic efficacy and maintaining high compatibility with bone and cartilage tissues (11).

### 3.3 Polymeric nanomaterials

Polymeric nanomaterials form one of the most flexible and biocompatible materials available for orthopedic use. They are synthetic polymers such as PLGA, PEG, and PCL, and natural polymers, such as chitosan, collagen, and hyaluronic acid (42). Polymeric nanoparticles are also widely used in drug and gene delivery because of their ability to encapsulate a wide range of therapeutic molecules and deliver them in a controlled and sustained manner (43). Chitosan nanoparticles bind effectively to cartilage surfaces and improve drug retention in the joint (44). In contrast, PLGA nanoparticles are normally used to deliver anti-inflammatory drugs, growth factors, and siRNA to osteoarthritic pathways (45). Polymeric nanomaterials are highly effective in long-term orthopedic treatments because their degradation rates are tunable and they are also not toxic (46).

### 3.4 Carbon-based nanomaterials

Carbon nanotubes (CNTs), graphene oxide (GO), and carbon quantum dots are examples of carbon-based nanomaterials with outstanding mechanical strength, electrical conductivity, and high surface area, which makes them very useful in orthopedic tissue engineering (47, 48). Carbon nanotubes assist in adhesion and differentiation of mesenchymal stem cells into chondrocytes and osteoblasts, whereas graphene oxide improves the mechanical stability of hydrogels and scaffolds in cartilage repair (49). Drugs can also be delivered using these materials, and they can interact with cells at the molecular scale to modulate signalling pathways involved in bone and cartilage regeneration. Carbon-based nanomaterials are especially helpful in the enhancement of scaffolds, cell growth, and functional scaffolds to be used in therapeutic delivery due to their distinctive physical and structural features (50).

### 3.5 Nanocomposites

Composite nanomaterials are nanomaterials that unite two or more different kinds of nanomaterials to get a stronger and more multifunctional effect (51). Examples include hydroxyapatite-polymer composites for repairing bone-cartilage interfaces (52), graphene-polymer hybrids to enhance scaffold strength, and metal-ceramic composites to enhance antimicrobial and osteogenic properties (52). Comprising the merits of each constituent material, i.e., the biopotency of ceramics, the stretchability of polymers, or the mechanical support of carbon-based structures, the composite nanomaterials can be used to achieve better performance in orthopedic treatment (53). They can effectively stimulate bone growth, deliver drugs, enhance mechanical strength, and reduce inflammation, which is why they are among the most promising materials for treating advanced osteoarthritis (54).

## 4 Application of nanomaterials in orthopedic therapy

### 4.1 Bone regeneration

**Figure 2:**
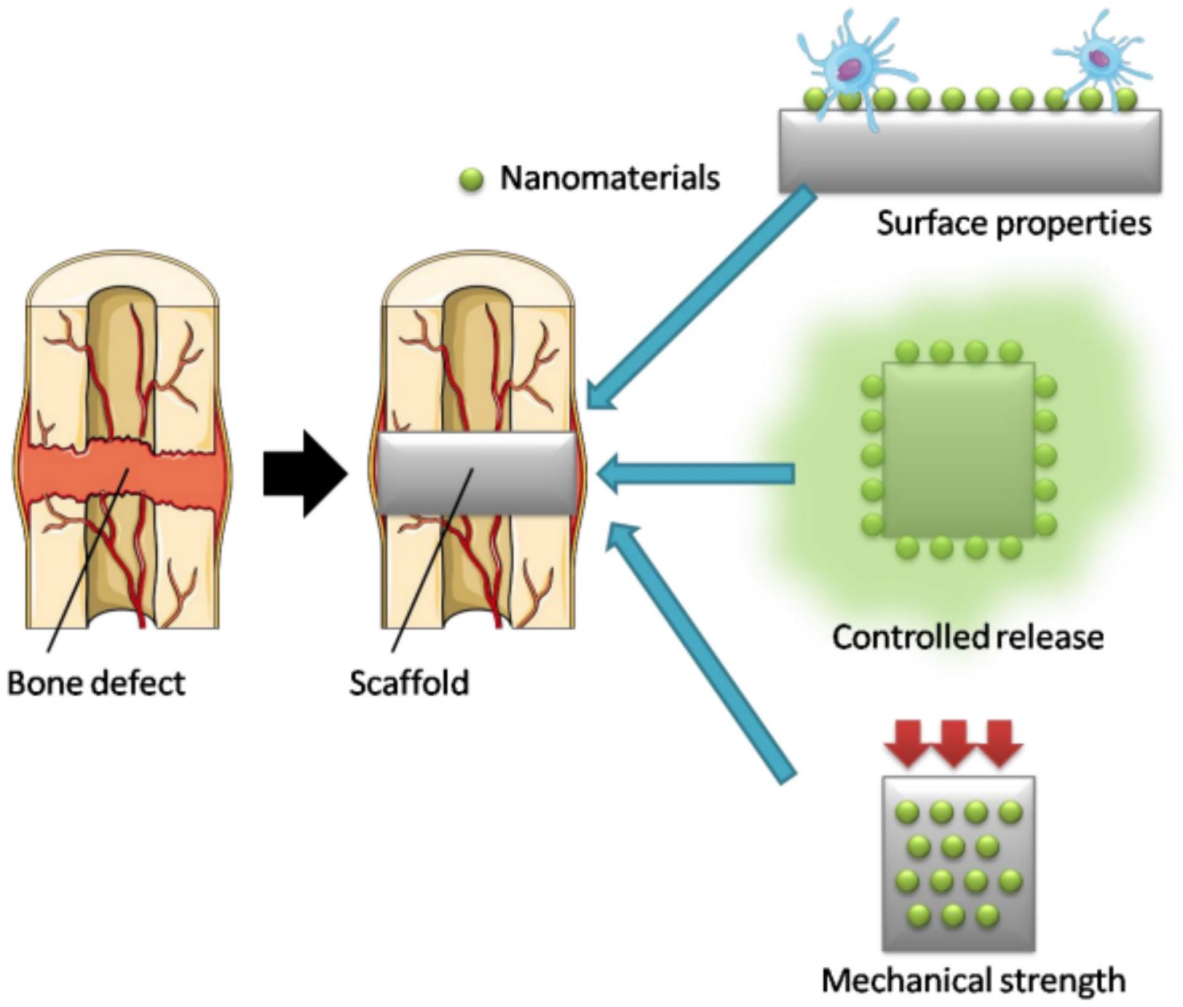
Role of nanomaterials in bone regeneration. Nanomaterial-modified scaffolds enhance surface properties, mechanical strength, and controlled release of therapeutic agents to improve the healing of bone defects. Reprinted with permission from (73) by the authors Tanaka M, Izumiya M, Haniu H, Ueda K, Ma C, Ueshiba K, et al. Licensee MDPI, Basel, Switzerland. This article is an open access article distributed under the terms and conditions of the Creative Commons Attribution (CC BY) license.

Nanomaterials have been important in bone regeneration since they have nanoscale properties that have a strong resemblance to the natural bone extracellular matrix, which consists of collagen fibers and hydroxyapatite crystals. Hydroxyapatite, bioglass, calcium phosphate, and metal-polymer composites are also nanoparticles that give good platforms on which new bone develops (55). They facilitate the adhesion, proliferation, and differentiation of osteoblasts that are required in bone healing. Mechanical strength and bioactivity of nanostructured scaffolds are also superior to those of traditional materials, enabling bridging of bone defects and healing of subchondral bone damage that is typically present in osteoarthritis (9). Also, osteogenic growth factors (e.g., BMP, VEGF, IGF-1) can be delivered in controlled-release format using nanomaterials to increase bone regeneration with minimum side effects. The hierarchical structure of bone can be readily mimicked by them, which is why they are most useful for accelerating recovery and improving overall bone quality (56). Figure 2 shows the effect of nanomaterials on bone regeneration by enhancing the performance of the implanted scaffolds. When a bone defect receives a scaffold, the surface of the scaffold is changed by incorporating nanomaterials, which enhance cell attachment and biological response (57). Additionally, nanomaterials enable the release of therapeutic agents from the scaffold and enhance its mechanical stability, thereby resulting in more efficient and longer-term bone healing (58).

### 4.2 Cartilage repair

The avascular and low-cellularity character of the cartilage tissue makes repairing cartilage quite difficult, and nanomaterials have provided new opportunities to execute an effective cartilage healing (59). Nanofibers, hydrogels, and composite scaffolds are biomimetic, recreating the cartilage extracellular matrix, and support chondrocyte survival, as well as increasing the deposition of collagen type II and proteoglycans. Chondrogenic factors like TGF-*β*, IGF-1, or certain microRNAs that induce stem cells to develop into chondrocytes can also be delivered by nanoparticles (60). Graphene oxide and Carbon nanotubes, as well as polymeric nanomaterials, enhance the mechanical strength of cartilage scaffolds, enabling them to resist compressive forces in the joint (61). Moreover, certain nanomaterials exhibit antioxidant or anti-inflammatory effects that protect chondrocytes from oxidative stress, a primary cause of cartilage destruction in osteoarthritis. These functions allow nanomaterials to play a great role in cartilage regeneration, as well as in the restoration of joint functionality (62).

### 4.3 Implants coating and surface modifications

Nanomaterials are extensively utilized to coat orthopedic implants to enhance their functionality, durability, and acceptance by the neighboring tissues. Nanosstructured surfaces enhance surface roughness and osteoblast adhesion, resulting in stronger and faster osseointegration. This is particularly crucial where the implants are joint replacements, screws, plates, and dental implants (63). The use of metallic type nanoparticles such as silver, copper, and zinc oxide in the coating of implants is common due to their high antimicrobial activity, which lowers the chances of infection and biofilm formation during post-surgery (64). Titanium dioxide nanocoatings are also used to enhance corrosion resistance and bone-implant adhesion. Also, nanomaterial coatings could target anti-inflammatory or osteogenic drugs to the implant site and prevent inflammation and induce bone growth in the area. These advancements improve the stability of the implants, decrease failures, and improve long-term orthopedic surgery (65).

### 4.4 Drug and gene delivery

Nanomaterials have become potent vectors for transferring drugs, genes, and biologics to the target affected tissues in orthopedic diseases, particularly osteoarthritis. Anti-inflammatory, analgesic, antioxidant, growth factor, or nucleic acid (siRNA, miRNA) can be encapsulated in polymeric nanoparticles, lipid-based nanocarriers, dendrimers, or exosome-inspired nanovesicles (66). They can be absorbed in cartilage tissue more efficiently than traditional drug formulations due to their nanoscale size, to provide deeper penetration and a prolonged retention in the joint. Nanocarriers preserve therapeutic agents and enable slow release into the body, thereby extending the dosing interval to days or weeks and reducing systemic side effects to the extent possible (67). Nanomaterials in gene therapy can be used to deliver genetic material to silence inflammatory pathways or stimulate cartilage regeneration by upregulation of regeneration genes. Magnetic or targeted nanoparticles may also be used to improve precision, in which drugs are only targeted to affected regions. In general, drug and gene delivery systems based on nanomaterials have a better therapeutic efficacy and are one of the most promising and developed in orthopedic nanomedicine (68, 76).

## 5 Challenges and limitations

Although nanomaterials are highly promising in orthopedic therapy, they have a number of challenges and limitations preventing their common clinical implementation. Among the most urgent issues to be addressed is the question of safety and biocompatibility, given the limited understanding of the long-term consequences of many nanomaterials. Certain metallic nanoparticles can affect the tissues or organs, accumulating and causing toxicity, oxidative stress, or chronic inflammation. Safe degradation and clearance from the body are critical, but a significant challenge to science. Stability and aggregation are another form of challenge (69). Nanoparticles can aggregate under physiological conditions, such as in synovial fluid, reducing their therapeutic activity and altering their biological interactions. This instability may also impact their capacity to penetrate cartilage and the prolonged release of drugs. In addition, their clinical application has been complicated by immune responses and interpatient variability, with the immune system capable of detecting and destroying nanoparticles before they can exert therapeutic effects (67, 77).

The major obstacle to advancement is the multifactorial, complex pathophysiology of osteoarthritis. Multifunctional designs are more costly and technologically challenging to engineer because OA is not driven by a single factor; therefore, a monofunctional nanomaterial may not yield a significant clinical outcome. Additionally, there is the issue of translating laboratory results into clinical use, which poses difficulties in upscaling production and maintaining uniformity, reproducibility, and sterility (70). Regulatory organizations like the FDA are struggling to assess nanomedicines due to the fact that current structures were structured to test conventional drugs and biomaterials. Consequently, the process of regulatory approvals is time-consuming, expensive, and unpredictable. Lastly, it has not been widely studied in human clinical trials; most studies on nanomaterials remain in the preclinical phase. Lack of clinical adoption is due to the lack of real-world information on long-term outcomes, biodegradation, and safety (71). These challenges have to be dealt with through more rigorous scrutiny of the safety of the products, standardization of manufacturing procedures, interdisciplinary cooperation, and effective regulatory procedures so that nanotechnology can proceed safely in the field of orthopedic therapy (23).

## 6 Conclusion and future perspectives

Nanomaterials have become potent technologies in orthopedic treatment, whose primary benefits are inaccessible to conventional materials and treatment methods. Their nanoscale size, high surface area, and tunable physicochemical properties enable them to deliver drugs directly to target sites, promote cartilage and bone regeneration, and improve implant integration. These functions overcome significant shortcomings in the management of osteoarthritis that include the inability of drugs to remain in the joint, tissue healing capacity, and problems related to orthopedic implants. Many studies have shown that metallic, polymeric, ceramic, carbon-based, and composite nanomaterials can control inflammation, enhance extracellular matrix formation and cell differentiation, and thus are widely applicable as therapeutic agents.

In the future, new strategies that combine nanomaterials with cell-based approaches are becoming increasingly popular. Nanostructured scaffolds with potential to facilitate cell adhesion, cell survival, and cell differentiation, along with biomaterials that control cellular microenvironment at the local level, may also be used to improve cartilage and sub-chondral bone regeneration. Moreover, the use of nanomaterials to facilitate the delivery of cell-derived bioactive factors, including exosomes and other extracellular vesicles, is a promising indirect approach to cell-based osteoarthritis treatment.

Despite these developments, there remain significant obstacles to the clinical translation of nanotechnology-based orthopedic therapies. Issues related to long-term biocompatibility, biodistribution, toxicity, large-scale manufacturing, and regulatory acceptance remain to be discussed. In addition, osteoarthritis is a multifactorial disease that necessitates multifunctional nanoplatforms capable of addressing inflammation, oxidative stress, and tissue degeneration simultaneously. The prospects involve designing safer, biomimetic nanomaterials, intelligent drug delivery, and nanotechnology stem cell therapies. To transition these technologies into clinical use, it is necessary to strengthen preclinical assessment, further clinical trials, and develop clear regulatory guidelines. Nanotechnology has a vast potential to revolutionize the way orthopedic and osteoarthritis are treated through further innovation and interdisciplinary cooperation.
